# A Survival Guide for the Rapid Transition to a Fully Digital Workflow: The “Caltagirone Example”

**DOI:** 10.3390/diagnostics11101916

**Published:** 2021-10-16

**Authors:** Filippo Fraggetta, Alessandro Caputo, Rosa Guglielmino, Maria Giovanna Pellegrino, Giampaolo Runza, Vincenzo L'Imperio

**Affiliations:** 1 Pathology Unit, ASP Catania, “Gravina” Hospital, 95041 Caltagirone, Italy; rosa.guglielmino@aspct.it; 2 Department of Medicine and Surgery, University of Salerno, 84121 Salerno, Italy; alessandro.caputo94@gmail.com; 3 Health Care Management Unit, ASP Catania, “Gravina” Hospital, 95041 Caltagirone, Italy; mgiovanna.pellegrino@aspct.it; 4 Superintendency Unit, ASP Catania, “Gravina” Hospital, 95041 Caltagirone, Italy; giampaolo.runza@aspct.it; 5 Pathology, Department of Medicine and Surgery, ASST Monza, University of Milano-Bicocca, 20900 Monza, Italy; vincenzo.limperio@gmail.com

**Keywords:** digital pathology, WSI, LIS, 2D-barcode, primary diagnosis

## Abstract

Digital pathology for the routine assessment of cases for primary diagnosis has been implemented by few laboratories worldwide. The Gravina Hospital in Caltagirone (Sicily, Italy), which collects cases from 7 different hospitals distributed in the Catania area, converted the entire workflow to digital starting from 2019. Before the transition, the Caltagirone pathology laboratory was characterized by a non-tracked workflow, based on paper requests, hand-written blocks and slides, as well as manual assembling and delivering of the cases and glass slides to the pathologists. Moreover, the arrangement of the spaces and offices in the department was illogical and under-productive for the linearity of the workflow. For these reasons, an adequate 2D barcode system for tracking purposes, the redistribution of the spaces inside the laboratory and the implementation of the whole-slide imaging (WSI) technology based on a laboratory information system (LIS)-centric approach were adopted as a needed prerequisite to switch to a digital workflow. The adoption of a dedicated connection for transfer of clinical and administrative data between different software and interfaces using an internationally recognised standard (Health Level 7, HL7) in the pathology department further facilitated the transition, helping in the integration of the LIS with WSI scanners. As per previous reports, the components and devices chosen for the pathologists’ workstations did not significantly impact on the WSI-based reporting phase in primary histological diagnosis. An analysis of all the steps of this transition has been made retrospectively to provide a useful “handy” guide to lead the digital transition of “analog”, non-tracked pathology laboratories following the experience of the Caltagirone pathology department. Following the step-by-step instructions, the implementation of a paperless routine with more standardized and safe processes, the possibility to manage the priority of the cases and to implement artificial intelligence (AI) tools are no more an utopia for every “analog” pathology department.

## 1. Introduction

A progressively increasing number of pathology departments are deploying, or planning to deploy, digital pathology systems for all or part of their diagnostic output [1,2,3,4,5]. Some authors already experienced the full transition to a digital workflow [6], eventually upgrading the scanning procedures at the magnification of 40× and even integrating artificial intelligence (AI) tools for the assessment of specific specimens (e.g., prostate biopsies) in routine practice [7]. Moreover, the employment of a secure virtual private network (VPN) connection allowed pathologists to work off-site [8], significantly helping during the recent COVID-19 pandemic [9].

However, despite this revolutionary transition, real world data suggest that a fully digital approach to the histological workflow has been implemented in only a minority of pathology laboratories, in Italy as well as worldwide. Several reasons have been advocated to explain what is holding us to the traditional “analog” workflow [10]. Although some major benefits of the digital approach (e.g., safety, quality, efficiency, easy and equal access to expert pathologists/second opinions) are widely recognized, some points may still cause the reluctance of the pathology community, starting from the costs, the lack of validation data and the possible “threat” represented by this kind of implementation for the pathologists [11].

Moreover, the Food and Drug Administration (FDA) approved some but not all of the available scanning systems (Philips and Leica) for digital primary diagnosis. The current lack of approval for all the other devices (e.g., 3DHistech, Hamamatsu, Ventana, etc.) is further slowing down the transition, even in the United States where a widespread implementation of a fully digital workflow using WSI for primary diagnosis is still in progress.

All these components contribute to the generalized skepticism of the pathologists towards these innovative paradigms, at least partly explaining the slow implementation of digital pathology in routine. The adoption of the advocated workflow is further complicated by the substantial lack of an adequate tracking system based on linear or 2D barcodes in the majority of the laboratories, which could represent an obstacle to benefit from all the advantages of the digital transition [11].

Based on the previously reported “Catania” experience at the Cannizzaro Hospital [6], this paper shows the step-by-step process followed by the Pathology department of the Gravina Hospital in Caltagirone (Sicily, Italy) to switch from a non-tracked system to a fully digital workflow in a few months, fully embracing all the benefits of digital pathology.

This may exemplify a simple and efficient transition from the glass slides to the WSI, thanks to the logical implementations made in the Pathology Laboratory of Caltagirone, in which the introduction of slide scanners represents only the last intuitive step of a complete digital workflow. Our experience is reported to the benefit of the numerous laboratories planning or working to implement digital pathology (DP).

## 2. Materials and Methods

**Figure 1:**
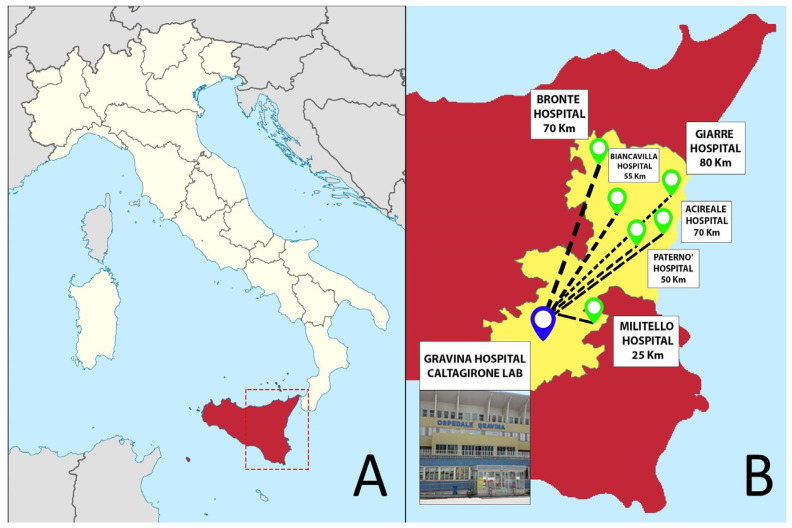
(**A**), Location of the Catania area in Sicily, south of Italy. (**B**), the different hospitals in the Catania territory referring to the Caltagirone pathology laboratory at Gravina Hospital.

The Gravina Hospital represents the Pathology laboratory hub of the Azienda Sanitaria Provinciale (ASP) of Catania in Sicily (south of Italy), collecting specimens—mainly surgical and bioptic samples—from 7 different hospitals distributed in the Catania area (Figure 1). Starting from 2019, the pathology department of Caltagirone experienced a profound transformation that required about 4 months to switch from a non-tracked, “conventional” pathology workflow to a fully digital approach. Similar to the previous “Catania experience” [6], the entire workflow was converted into a digital one, but introducing some additional “digital” checkpoints through the different steps of the process. 

To allow and facilitate this transition, the following implementations were needed:

1. Lean workflow and rearrangement of spaces and offices;
2. Implementation of the information technology infrastructure;
3. Implementation of the tracking system and checkpoint procedures;
4. Implementation of the automation;
5. Implementation of the scanning.

### 2.1. Lean Workflow and Rearrangement of Spaces and Offices

In order to achieve the best result of the digitization we first solved some logistic problems in the lab. Following the Lean approach philosophy [12,13], the spaces were rearranged: this started from a redistribution of the rooms in a linear manner based on the natural sequence of the sample processing steps. This significantly reduced the personnel and specimen transfers and optimized the working time through the arrangement of similar tasks (e.g., staining and scanning) in the same room and through the creation of inter-room communications. Thanks to a better distribution of the spaces, these modifications freed two rooms that were used to create the molecular section, previously absent in the lab partly due to the inefficient disposition of the spaces.

### 2.2. Implementation of the Information Technology Infrastructure

Before the implementation of the digital workflow, a dedicated network as well as servers to store the images and the linked metadata were lacking in the lab. As a consequence, the spaces and offices were not equipped with the necessary access points for the network, and the different instruments used for the analog workflow were not interconnected through the laboratory information system (LIS). Thus, along with the adoption of a Lean approach to the workflow, we implemented the information technology infrastructure: this consisted in the creation of internet access points (network access) based on the position and type of instruments to be connected and a dedicated bandwidth of 100 Mbps.

**Figure 2:**
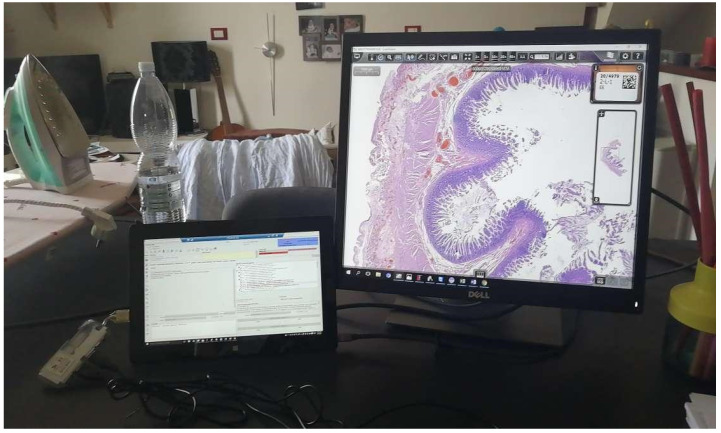
One of the “home-made” working stations used by pathologists for the off-site sign-out and reporting. The smaller monitor (on the left) has a sufficient size and resolution to run the LIS. The right display allows an adequate visualization of the WSI.

The entire digital workflow switch has been centered on the implementation of an anatomic pathology LIS (AP-LIS), Pathox (version 13.22.0, Tesi Elettronica e Sistemi Informativi S.P.A., Milan, Italy), allowing the integration of the case/sample information from the accessioning to the reporting phases. The majority of the instruments present in the lab were integrated with the LIS using the 2D barcode system with interface exchanges handled through Health Level 7 (HL7) version 2.5 messages. Based on the previous experience [6], the integration took only a few days of work (including the implementation of the scanner which took 2 days). This is in contrast to other reported similar implementations that required more time to deploy [14,15]. Furthermore, the implementation of a secure VPN connection allowed the pathologists to access and report cases from home (Figure 2). 

### 2.3. Implementation of the Tracking System and Checkpoint Procedures

The Lab lacked a proper tracking system and tissue blocks as well as glass slides were handwritten. Not all the steps of the workflow were appropriately tracked (i.e., gross examination, tissue processing and paraffin-embedding) and different/redundant paper sheets accompanied the workflow from the accessioning to the assembling and delivering of the glass slides for each phase. This “analog” workflow was abandoned in favor of a new paperless 2D-barcode tracking system, fully integrated with the LIS. 

This new system was then implemented through the entire workflow, from accessioning to diagnosis. 2D barcodes were preferred to 1D ones because they are less space-demanding (fitting well on the tiny surface of both tissue blocks and glass slides), more easily applicable to the convex surfaces of tissue containers, and generally less prone to scanning issues. Moreover, we introduced laser printers for blocks in order to obtain a permanent mark of the barcode on the surfaces (See Grossing section of the Results). The implementation of the tracking system within the LIS gave us the possibility to monitor safely and efficiently every step of the workflow through the use of dashboards. 

### 2.4. Implementation of the Automation

To promote the automation of the process following the Lean philosophy, some instrumental implementations were introduced in the laboratory, simplifying many laboratory procedures that were previously performed manually and in a repetitive manner (Table 1). All of these achievements were made possible mainly thanks to the HL7 connection and the widespread use of 2D barcodes. However, the prototype of automation was represented by the automatic assembling and delivering of the slides through the use of scanning systems together with the 2D barcode–based archiving of blocks and slides. Finally, the immunohistochemistry instrument (Autostainer Link 48, Agilent, Santa Clara, CA, USA) was completely interconnected with the LIS (Pathox) through the HL7 connection.

### 2.5. Imaging Technology

Since the main paradigm chosen for the digital workflow switch was based on the LIS-centric philosophy, this allowed a perfect integration of different scanning platforms independently from the vendor, the WSI formats (e.g., .tiff, .svs, .vms, .ndpi) and the provided platform for slides visualization. This change in the paradigm did not force the department to employ a specific scanner device, leading to choose a fast (35 s/slide) and high throughput (60 slides/h) scanner (Pannoramic 250 flash III, 3DHistech, Budapest, Hungary), with a load capacity of 300 slides and good performances with brightfield and darkfield applications. The digitization involved standard hematoxylin and eosin (H&E), special histochemical, immunohistochemical and immunofluorescence slides (for both conventional immunofluorescence and fluorescence in situ hybridization, FISH). For the frozen sections and intraoperative procedures, the Aperio LV1 IVD system (Leica Biosystem, Nussloch, Germany) was employed due to the ability to obtain live images from up to 4 slides with magnification up to 63×. Digitizing cytology slides was not undertaken due to the need for Z-stack image acquisition which increases scan time and file size [16]. The scanning system was operated by technicians who were trained to use these devices to support routine daily work. To further optimize the workflow, the scanning station was located in the same room where slides were stained, coverslipped, and prepared for archiviation (Stainer AUS 240, Bio-optica, Milan, Italy; Leica Coverslipper CV5030, Leica Biosystems, Nussloch, Germany). Regular maintenance was performed every month taking into account white/color balance and adjustment of the scanner focus.

After the scanning process, the slides were automatically assigned to the proper cases and “virtually” delivered to the pathologists [6]. The slides appeared in the “virtual tray” within the LIS and cases with scanning completed for all the slides belonging to them were considered ready to be reported. 

Pathologists’ workstations were composed of one computer with 2 monitors. Different computer devices have been implemented for the pathologists’ workstations (Table 2), with central processor units (CPU) of different generations, different clock speed and vendors (Intel and AMD), random access memory (RAM) with different size (4 and 8 GB) as well as various video cards, mostly integrated.

Two monitors with different roles have been connected to each computer, allowing the simultaneous evaluation of the case-page in the LIS and the respective WSI from different displays. As per manufacturer instructions, the employed LIS required a minimum of 17 inches monitor to run, and the department introduced devices with a range of 17–27 inches. On the other hand, the monitors dedicated to the visualization of the WSI were 24–27 inches in size (Table 3). 

WSIs were directly accessed from the AP-LIS. Specifically, a virtual slide tray was created and incorporated within the AP-LIS, as already described [6]. Accessioning of cases and real-time tracking of digital slides occurred directly from the AP-LIS. The creation of a single slide tray within the AP-LIS Pathox, displaying the macroimage (thumbnail) of several slides simultaneously, allowed the incorporation of WSI acquired from the Pannoramic 250 Flash III scanner, with the possibility to connect different scanners from different vendors (by using the image management system of the scanner as a simple middleware) without disrupting the end-user workflow. All images were saved on network-attached storage (96 TB Qnap NAS TVS-EC1280U-SAS-RP) using the dedicated 100 Mbps network connection. All the scanned slides are stored in the server as a digital database of WSIs, allowing a possible retrospective consultation directly from the AP-LIS. The eventual re-scan of a slide resulted in the overwriting of the previously scanned one, so that pathologists always had access to the most recent images. Validation of the WSIs for their use for primary histological diagnosis was made according to the CAP guidelines [17].

## 3. Results

In 2019, just before the advent of COVID19, the Caltagirone pathology department had a yearly workload of 8182 histological cases with a total of 42,245 corresponding slides. The entire activity of the laboratory has been modified starting from the limitations and issues related to the previous “analog” and non-tracked workflow, following different steps (checkpoints), as reported below. This allowed a complete transition towards a digital pathology approach, leading to the digital primary sign-out of all the cases through WSIs. Before the implementation of the digital workflow, no standard procedures and checkpoints were present along the different steps of sample processing. Addressing these deficiencies was mandatory for the full digital transition, in order to have a more efficient fully tracked and paperless workflow. Here we report the introduced checkpoints at every step of the specimen handling that should be followed to obtain a fully integrated system (Table 4).

### 3.1. Accessioning

Before: specimens were sent to the Pathology Lab of Caltagirone on specific days from different hospitals, accompanied by a request without an order entry. During the accessioning phase, a progressive number was created along with an additional internal paper (lab sheet), used later on as the working paper for the subsequent phases. 

After: the creation of an appropriate checkpoint at this step allowed the laboratory personnel to complete these accessioning tasks in an unbiased way to minimize the risk of errors. To reach this aim we employed a combination of barcode printer and reader, as well as the introduction of a paper flat scanner (A4 format). These technologies helped in the univocal identification of the case/sample/patient from the accessioning phases (through the 2D barcode printer/reader), adopting an order entry that facilitates the tracking system fully integrated with the LIS. The implementation of the order entry gave us the possibility to monitor the upcoming material from the different hospitals. Moreover, the availability of scanned documentation linked to the case allowed the pathologist a rapid consultation of all the sources needed in a paperless way. By introducing these procedures (order entry and possibility to scan all the documents) the accessioning errors dropped from 6.3% to less than 0.5% of the cases, as expected [18,19].

### 3.2. Grossing

**Figure 3:**
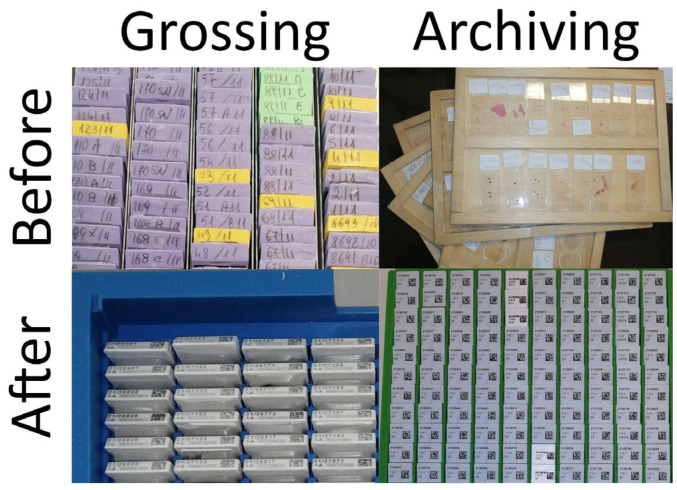
Comparison of some of the principal checkpoints before and after the implementation of DP tools. On the left, during the grossing phase the case identification number was handwritten on every cassette before the introduction of case-specific 2D-barcodes directly generated by the LIS and laser-printed on the cassettes. Similarly, hand-labeled glass slides were randomly returned to the technicians and manually archived (right). The introduction of WSI and scanner next to the staining instrument allowed the direct archiving of physical glass slides using the 2D barcodes.

Before: cases were sent to the grossing room with the generated working paper. Here, the sample grossing was performed by a pathologist with the support of a technician and the macroscopic description was handwritten on the lab sheet, with obvious consequent transcription and interpretation errors. Moreover, since no barcodes were used and the cassettes/blocks generated during this phase were handwritten, the risk of possible subsequent errors was further amplified (Figure 3).

**Figure 4:**
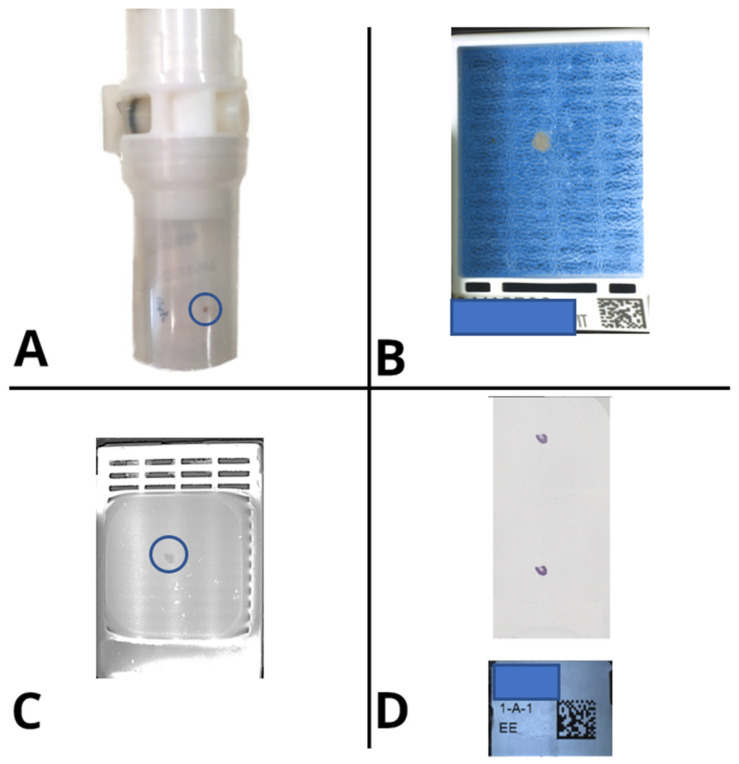
Digital pictures taken at each step of the life of the specimen and respective cassettes fully document the flow of tissue in the lab, allowing global traceability and high-resolution error tracking. (**A**) Specimen container as it is received; (**B**) Cassette at grossing, before closing its lid; (**C**) Surface of the FFPE block after microtome sectioning; (**D**) Macro picture of the glass slide after staining.

After: the routine grossing practice radically changed starting from the introduction of a barcode reader, leading to univocal recall of the correct case in the LIS by the pathologist after scanning the specimen container. Moreover, a digital camera (MacroPATHOX, Tesi Elettronica e Sistemi Informativi S.P.A., Milan, Italy) and a BlocDoc (SPOT Imaging, Sterling Heights, MI, USA) instrument were introduced in the room. The camera is used to take pictures of the specimen as it is received (before any sectioning has taken place) and then additional pictures are taken after sectioning to document macroscopic features such as tumor size and depth of infiltration. The pictures can be marked up to identify where the samples have been taken. This allows connecting each block—and thus each WSI—to its original anatomic location. BlocDoc is used to document sampling: each cassette is photographed before its lid is closed [20]. This serves as the reference standard for each block, to be compared with the other pictures which are taken post-processing, post-microtome sectioning, as well as the slide macro and WSI pictures (Figure 4), and is of crucial importance for surgical and bioptic samples alike. This significantly reduced the risk of losing precious material, creating a back-up of information useful to cross-check the adequacy of the specimen in the subsequent steps. Furthermore, inconsistencies can be traced back to the specific moment in which they happened (Figure 4). Finally, the employment of a laser printer allowed the automatic production of barcoded cassettes, further reducing the rate of errors during the subsequent phases (Figure 3). 

### 3.3. Processing

Before: cassettes containing the specimens were sent to the processing room, manually checking that all the cassettes generated during the grossing step are present in the rack that is going to be processed.

**Figure 5:**
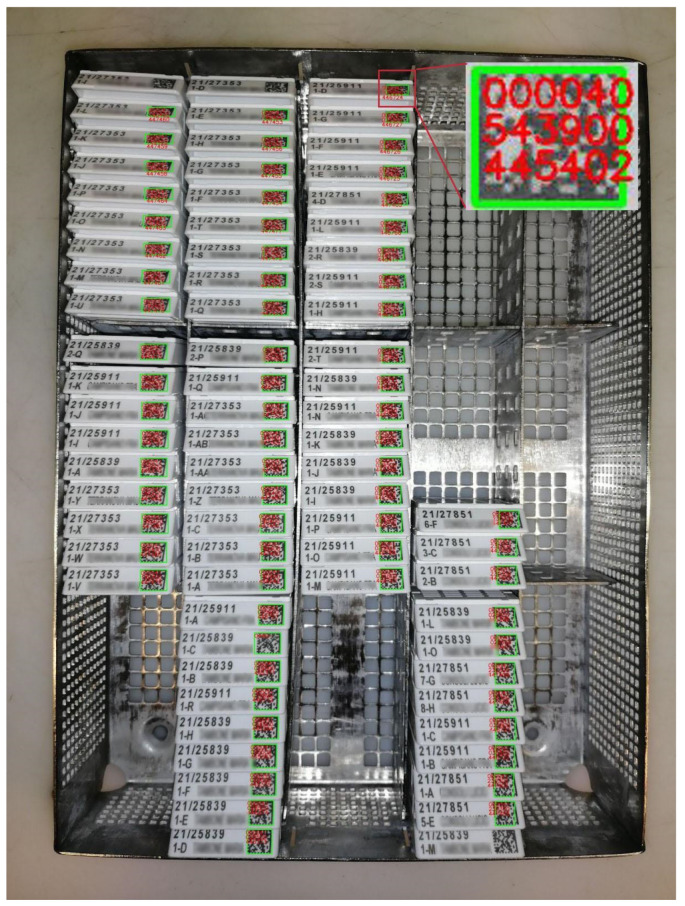
The reading process of barcodes directly from the rack containing the blocks during the processing phase after the digital transition. In the upper right inset the code extracted from the 2D barcode directly in the LIS.

After: through the employment of barcode readers, the entire rack is scanned in one go (with a single picture) before it is processed and a check is performed to verify that all the produced cassettes are submitted to the subsequent phase, thanks to the integration with the LIS. The presence of a dedicated dashboard within the LIS, showing all the blocks produced during the current grossing session, allowed us to implement an automatic check. At the moment there are several instruments in the market capable of reading all the barcodes in short time matching the cassettes present in the rack with those produced at grossing. In the Caltagirone pathology lab, the implementation of MacroPATHOX allowed the scan of all the produced blocks directly from the rack (Figure 5), matching the material sent to the processing room with the specimens produced by the grossing operator.

### 3.4. Embedding

Before: technicians embedded all the material found inside the cassettes without the possibility to verify the integrity of the specimen after the grossing and processing phases, with the eventual risk of losing material along the workflow especially in cases characterized by multiple small fragments of tissue. 

**Figure 6:**
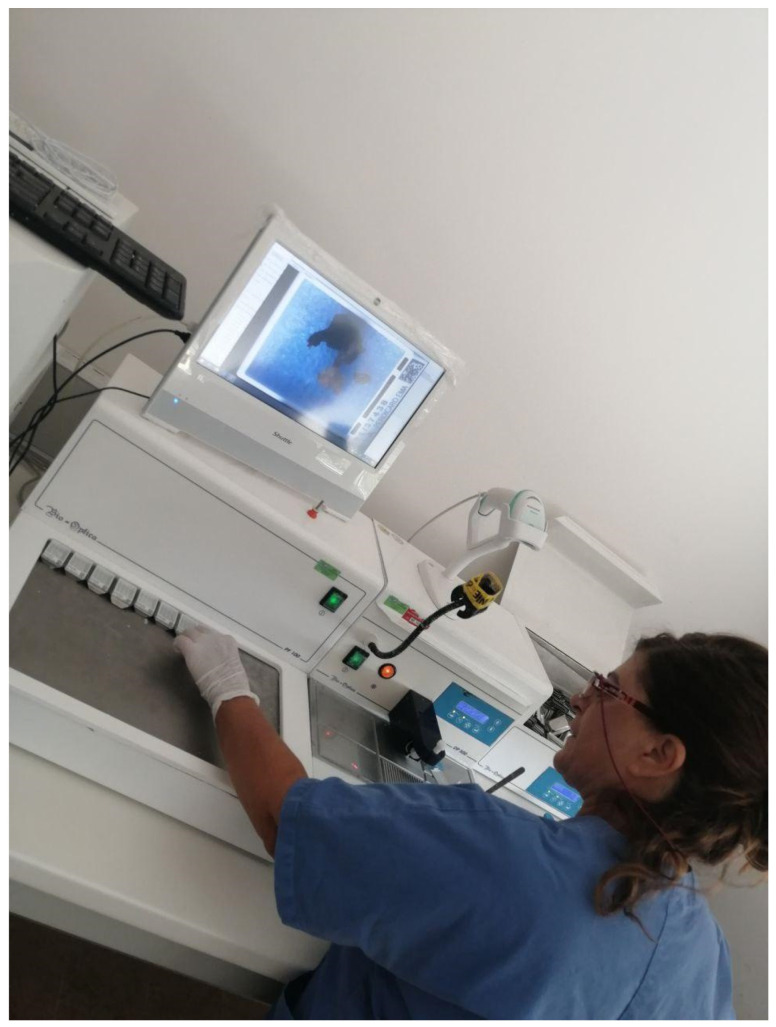
The BlocDoc at the embedding phase.

After: this issue has been solved by the availability of photographic documentation obtained in the grossing room, directly available for consultation from the case page in the LIS by the technician who can compare what was submitted by the pathologist with what is actually present in the cassette at the embedding station. Moreover, a correct embedding may prevent poor-quality slides from being produced and thus reduce scanning errors. Large fragments tend to be hydrated and may have a size difficult to be fully captured by the scanner. The adjustment of the size of the sample fragments must start at the grossing station and be verified during embedding. Similarly, well-oriented tissue fragments, levelled and close to each other in the paraffin lead to a better-quality glass slide. Finally, the introduction of BlocDoc to capture the content of cassettes/blocks during the embedding phase can represent a further checkpoint step to control the workflow (Figure 6).

### 3.5. Sectioning

Before: blocks (handwritten) were consecutively positioned on the microtome, and sections were collected by the technicians with the corresponding number handwritten on the glass slide. However, this again exposes to possible risks of misidentification and case exchange that can be prevented by the introduction of appropriate checkpoints at this step of the workflow. 

**Figure 7:**
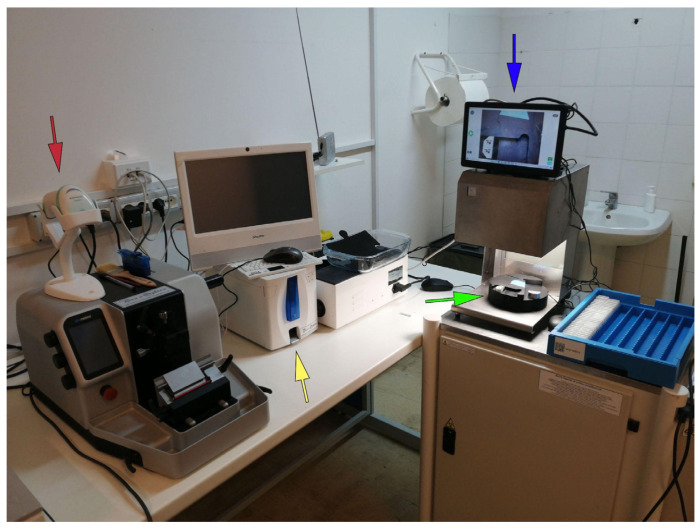
An example of a sectioning station. The technician can identify the block through a barcode reader (red arrow), entering the LIS page of the case and printing the related glass slides with a laser printer (yellow arrow). After sectioning, the technician can directly scan the cut surface positioning the block on the dedicated space in the BlocDoc instrument (green arrow), with the possibility to assess the preview of the obtained image (blue arrow).

After: a barcode reader has been added to every microtome station, allowing the technician to automatically identify the case and block directly on the LIS. Moreover, thanks to a slide printer, every operator now has the possibility to produce as many glass slides as required by the specific case without potentially error-prone human interference (i.e., handwriting). Finally, after the sectioning phase the cut surface of the block can be captured with an appropriate device “BlocDoc” (SPOT Imaging, Sterling Heights, Detroit, MI, USA) to obtain archival documentation that can be useful for the pathologist to assess the integrity of the material reported on the final virtual slide (Figure 7). The subsequent manual archival of the blocks is now substituted by a fully automated system based on 2D barcodes (Figure 5). 

### 3.6. Staining and Scanning

Before: the staining was manually performed, glasses were assembled on trays and delivered to the pathologists together with the accompanying lab sheet generated at the accessioning. The employment of differently colored cassettes indicated the need to perform special stains, as well as different types of samples or level of urgency.

After: Slides from the sectioning room are unequivocally and individually identified through the employment of a barcode reader. Thanks to the full LIS integration, this allowed to obtain all the needed information regarding the required stains transmitted by the simple barcode scanning without the need of human interpretation (e.g., of the colors of the cassette). The staining process moved from manual to automated by introduction of an automatic stainer (Bio-Optica, Milan, Italy) and it now follows the highest qualitative standards to minimize interferences with the scanning phase (faint or darker staining, debris/precipitates). For this purpose, the implementation of daily internal controls and/or external quality control can help in the assessment of the quality of stained slides [21]. After air-drying, stained and coverslipped glass slides are loaded into scanner slide racks and scanned. Up to 300 slides can be loaded at a time; the scanner can operate continuously and more racks can be loaded while it is running. Since the workflow was organized in the production of small batches in order to obtain a continuous workflow, the scanner was loaded with glass slides just a few hours after staining (as soon as they were dry) with a limited use of the overnight batch scanning session. Implementation of continuous workflow within the laboratory (i.e., cutting, staining, and then immediate scanning before signout activity) allowed the laboratory to achieve complete slide creation and digitization of all the produced slides within the same day. We observed a scan failure rate of approximately 0.5%, mostly due to problems in the recognition of the 2D barcode printed on the slide, and occasionally due to network connectivity problems. 

### 3.7. Final Reporting and WSI Viewing

Before: pathologists assessed glass slides using a microscope. The final report was not written directly into the LIS but manually transcribed on the lab sheet originally generated at accessioning. The further requirements for the diagnosis (e.g., special stains, immunohistochemistry, additional recuts) were handwritten and personally delivered by the technicians through the creation of a new internal lab sheet. Since no administrative personnel was available, all the information reported on the lab sheets were personally typed inside the former LIS at the end of the day. The slides used to render the diagnoses were randomly returned to the technicians to be archived (Figure 3). 

After: Today, the AP-LIS digitally presents a work list to the pathologist, as cases ready to be reported, urgent cases, cases waiting for additional cuts or additional staining, with clear indication of the presence of digital assets and/or pending status. Pathologists can then access each case from the work list and can open the respective virtual slides shown in the virtual tray with a double-click. WSIs appear on the dedicated monitor and are viewed using the original scanner viewing software. Moreover, the AP-LIS allows to make a direct and quick comparison with the gross specimen embedded in the paraffin block, thanks to the availability of BlocDoc (SPOT Imaging, Sterling Heights, Detroit, MI, USA) scans readily obtainable with a double-click on the tissue block entry in the “virtual tray”.

Documents (digitized by the A4 flat scanner at the accessioning), and macroscopic images taken at the grossing were all available with a simple click. This allows fine-grained error tracking and global traceability. For example, if a fragment is missing in the WSI, the pathologist can examine the request form and pictures of each stage of the tissue processing (Figure 4) to identify exactly what went wrong. For example, the fragment might be present on the slide but missed by the scanner’s tissue finder, or it may be embedded deep in the tissue block and require further sectioning to be analyzed. It may have been lost during processing, or it may have been missed at the grossing station, or it may not have been sent to the pathology lab at all (Figure 4). The pathologist can clearly identify what went wrong, when and where, without ever leaving his desk. Moreover, the final diagnosis could be rendered using the traditional narrative style or according to a well-defined synoptic report, optionally using guided checklists. A selection of images (gross or microscopic) can be included in the final report for clarity, directly from the LIS. 

### 3.8. Archiving and Retrieval of Tissue Blocks

Before: After microtome sectioning, (handwritten) tissue blocks were manually and painstakingly reordered and archived consecutively by case number.

Retrieval of a block entailed identifying the correct drawer (by case number), then searching for the position in the drawer where the block should be, and hoping to find it there. In case of missing blocks (archival errors due to misreading of the handwritten label, or blocks retrieved and never re-archived) there was no way to know where the block was, who took it, or where it was last seen. This process was lengthy and error-prone. Frequently, the glass slides were delivered to the pathologists before the respective tissue blocks had been archived. If the pathologist requested a special stain, a painstaking search for the block in the archive as well as in the sectioning room would ensue, adding friction and delays.

After: After microtome sectioning, each block is immediately stored in a random spot in a dedicated rack, barcode facing up. At the end of the sectioning session, the rack is photographed by a dedicated scanner which, thanks to its integration with the LIS, automatically marks each block as archived and logs the rack number and the coordinates within the rack, as well as the operator, date, and time.

Retrieval is fully automated and computer-guided. The operator who wants to retrieve a block is guided by a handheld personal digital assistant (PDA) to the correct rack, and then to the position within the rack where he will find the block. Upon withdrawal of the block, the action is logged and timestamped, and the operator is responsible for re-archival of the block. If the block is not in the archive (e.g., being recut for additional stains), the system will indicate who has taken the block and is responsible for its rearchival.

### 3.9. Archiving and Retrieval of Glass Slides

Before: After staining, coverslipping and air-drying, the technician was responsible for assembly and delivery of the case to the pathologist. Only after rendering the diagnosis, the slides were collected by the technician who had to regroup them and archive them manually.

After: After staining, coverslipping and air-drying, the slides are placed in the scanner racks with no particular attention to order. After scanning, virtual slides are stored in a dedicated database with a storage capability of 96 TB, and glass slides are archived in a dedicated rack in a random order, in a manner similar to blocks. The rack is then scanned and archived. The LIS receives data about each slide (rack number, position within the rack, as well as date and time of the archival and responsible operator).

Retrieval is fully automated and computer-guided, similar to tissue blocks.

### 3.10. Intraoperative Diagnosis Using Hybrid Instrument

In the Caltagirone example, a particular hybrid instrument (Leica LV1, Leica Biosystem, Nussloch, Germany) was chosen for its better performance in the live streaming of frozen-section slides and was located in the same room where the intraoperative procedures were performed (grossing room). This is in line with the lean redistribution of the spaces and offices which had been performed before the fully digital instrumentation was installed in the lab, thus logically allocating the scanning tools next to the staining facility. 

### 3.11. Molecular Pathology and Fluorescence

The rearrangement of the spaces and offices allowed the creation of an entire section of the laboratory dedicated to molecular pathology, previously absent in the department, introducing instrumentation for next-generation sequencing as well as for the “classic” genetic tests, such as real time PCR and FISH. The results of these exams were directly integrated in the case-page of the LIS, allowing the association of the standard histopathology report with the molecular characterization. Finally, the introduction of a scanner with optimal performances in darkfield applications (e.g., immunofluorescence), allowed the digitization of FISH samples that were directly associated to the case as any other WSI.

### 3.12. Computer-Aided Diagnosis (CAD) and Artificial Intelligence (AI) Tools

The deployment of the digital workflow gave the Caltagirone lab the opportunity to open the door to the third revolution in pathology, after the advent of IHC and genetics [22] through the introduction of artificial intelligence (AI) algorithms to aid the routine diagnostic assessment of cases. Although in a “futuristic” perspective some authors imagined a fully digital department in which all the cases/slides are presented to the pathologists after a first check performed by the AI algorithms, this has already been implemented in a first experience by one of the authors (FF) [6] and is now in the Caltagirone example an actual reality with a better concordance of diagnosis among pathologists when the AI tool Inify (Inify AI tool for prostate, Contextvision, Stockholm, Sweden) was used (92% vs. 98% concordance, personal data).

## 4. Discussion

In 2019 the Pathology department of Gravina Hospital in Caltagirone (Sicily, Italy) decided to start using digital slides for routine surgical pathology practice. The intent was to digitize all the histopathology glass slides, borrowing from the previous successful experience of Catania [6]. In this further example the digitization process was not merely limited to the “classic” paraffin block-derived slides (e.g., H&E, histochemical and immunohistochemical stains), extending the application to the fluorescence and frozen sections for intraoperative assessment as well. In the previous Catania experience the frozen sections were excluded from the scanning process due to logistic reasons and technical problems. To solve these issues, in the Caltagirone example a particular hybrid instrument (Leica LV1, Leica Biosystem, Nussloch, Germany ) was chosen for its better performances in the live streaming of frozen slides and was located in the same room where the intraoperative procedures were performed (grossing room). This is in line with a lean redistribution of the spaces and offices which has been performed before the full digital instrumentation was installed in the lab, helping in the logical location of the scanning tools next to the staining facility.

As suggested by the guidelines [17], a specific period of time was dedicated to validate the WSI as a substitute for the glass slides. The digital pathology system was deployed primarily to support clinical diagnostic work. Additionally, the implementation of this system allowed the access to WSIs directly from the LIS even during multidisciplinary team meetings or tumor boards [23]. The ability to work remotely was also made possible by the implementation of a secure VPN connection. 

The required training is far less than one might imagine. With computer-literate staff, training to use a new tool or machine does not take more than a short tutorial session (a few minutes to a few hours, depending on the tool) and 2–5 days to get used to it. Even for the most daunting things (e.g. the scanner, the LIS), complexity stems from the array of functions and settings, and not from the basic, everyday usage, which is surprisingly simple. The Caltagirone pathologists, as well as two of the authors (VL and AC) could proficiently load the scanner and launch a scanning job after a few minutes’ training. Similarly, they could confidently use the LIS for all the everyday functions on day two.

There is no dedicated group of individuals for scanning, image storage and digital infrastructure management. Each technician and each pathologist is taught how to perform basic tasks (e.g., loading, launching, and unloading the scanner) and is expected to be able to perform them.Despite the absence of pathology residents and trainees due to the non-academic nature of the Gravina Hospital, the digital transition allowed us to share anecdotal and didactic cases with young pathologists belonging to different residency programs in the Italian territory. This aspect stresses the invaluable educational role of digital pathology [24,25], especially during the recent COVID19 pandemic [26].

**Figure 8:**
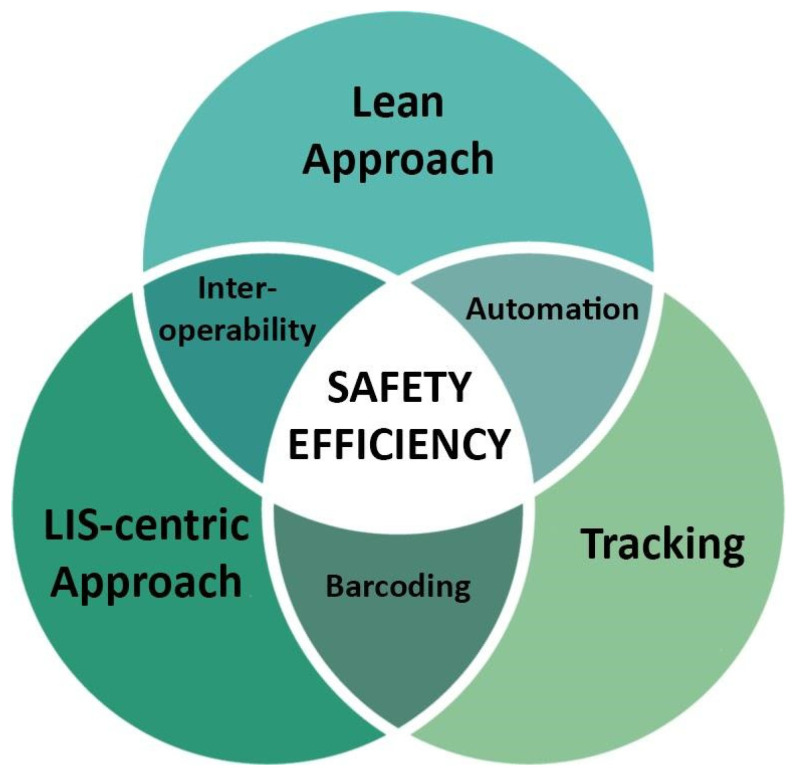
The importance and relationship of the main points required for the development of a reliable, sustainable and safe digital pathology workflow.

However, to fully benefit from the advantages of the digital transition, the process should follow a strict optimization of the resources, namely time, space, people and instruments, creating the conditions for increased efficiency and consequently decreased costs. Just like digital pathology is only incidentally about the slide scanner, the digital transition is only secondarily a matter of instruments. The transition must start from a strong leadership moving the entire group, motivating all the people to be game-changers. The transition then goes through the people, changes mindsets, workflows, and finally converges on new instruments. The Lean approach is an example of a strategy that can facilitate the management of the staff for the maintenance of turn-around time, starting from the most appropriate arrangement of the space which allows a more linear workflow, the reduction of disorganized sample traffic and thus the realization of a less time-consuming diagnostic process. Although different guidelines have been dedicated to describe the different steps needed for the digital transition [17,27], they mainly focused on the validation of the WSI tool without additional recommendations for the optimization of the pre-analytical steps. As clearly shown by the Caltagirone example, the implementation of digital pathology cannot be performed without a solid base consisting in a fully tracked anatomic pathology workflow (e.g., using the order entry and 2D-barcodes), the adoption of the Lean approach (e.g., through the employment of different automation instruments) and a fully integrated system with the AP-LIS (Figure 8). This triad further allows the interoperability of the different devices employed in the laboratory, independently from the vendor or the software interface adopted by every specific instrument, as demonstrated by the implementation of a fast and high throughput scanner without any compatibility problem. Moreover, the customization of the LIS led to the association of WSI deriving from different devices in the same slide tray, demonstrating the high versatility of this approach.

We are currently working to validate WSI in gynecological liquid-based cytology (LBC) using a Pannoramic P1000 scanner (3DHistech). Recently, new instruments dedicated to digitizing the LBC have appeared in the market, with the possibility to run AI tools to support diagnosis. Even when cytology slides were examined using a conventional light microscope, in spite of the absence of the “final” part of the digital transition (the WSI), numerous improvements had spilled over to the workflow of cytology cases. For example, these cases are fully tracked by the LIS (i.e. no paper worksheets) and benefit from the linearity, efficiency, and order of the lab.

Another crucial point that should be addressed before the digital transition is represented by the need of a dedicated, high speed network in the anatomic pathology laboratory (100 Mbps in the Caltagirone example), to prevent possible network issues that can potentially impair each automated phase of the process, from accessioning to sign-out [28]. Moreover, the availability of a dedicated storage system with an adequate capacity to allow the archiving of WSI is of paramount importance. In the present experience, a database of 96 TB has been employed, without a significant impact on the overall costs of the digital transition. Recently, more advanced solutions have been developed. For example, using the RAID 6 technology (redundant array of independent disks, level 6), one can implement a local storage solution with redundancy and back-ups at much lower costs than similar cloud-based solutions (approximately 10,000.00 € for 100 TB).

For the pathologists’ workstation requirements, some guidelines proposed the minimum prerequisites that the computers and monitors should have to be employed in the WSI visualization for primary diagnosis [29]. Despite these recommendations, different subsequent reports demonstrated the feasibility of digital sign-out of the cases (even in off-site settings) independently from the workstation solution chosen by each pathologist [30] and with a wide variety of combination of CPUs (1.3–3.2 GHz), monitors (13.3 to 25 in) and browsers employed [31]. As a further demonstration of the relatively low technological requirements for digital sign-out, in Caltagirone the introduction of workstations with different technical specifications did not significantly impact on the final quality of the WSIs as well as on the end-user experience. This was valid for home working as well, thanks to the secure VPN connection. In this case home computers and non-medical-grade devices were used, without significant impact on the final histological diagnosis. Remote working for pathologists is still a young and underdeveloped concept, but the COVID pandemic helped boost its adoption. During the lockdowns, more than half of the cases were signed out remotely, effectively reducing on-site work to the bare minimum (i.e., grossing, intraoperative exams, and cytology). Incommensurable individual and social risks were avoided thanks to remote work.

The implementation of a digital workflow contributes to increase the efficiency and safety of the different processing phases, through the introduction of specific checkpoints at every step, allowing a more adequate quality control from the accessioning to the final reporting (Figure 4). In the present experience, only a minority of slides had scanning issues, most commonly due to focus problems or in the tissue finding algorithm. For example, abundant white adipose tissue (as can be seen in lipomas) can sometimes be ignored by the tissue finder, or some parts of the slide might be out of focus. Some authors advocate for a routine check of all WSIs by the technician before delivery. In our experience, these errors are rare (<5%), often affect very small parts of the slide, and rarely cause diagnostic problems. For these reasons, assigning the manual check of each WSI scan to the technicians would lead to a significant time-consuming process for less than 5% of rescan. As an alternative solution, in the proposed workflow the pathologists can eventually order, if needed, a rescan directly from the LIS (as it would happen for an additional stain or ancillary tests). Efforts are underway to automate this quality check phase [17,18,19]. Furthermore, tissue coverage can be checked by the pathologist by comparing the WSI to the slide macro image. In this setting, the recent introduction of a specific instrument, namely BlocDoc, for the detection of tissue inconsistencies, further allowed to increase the accuracy of the technicians’ and pathologists’ work. It has been estimated that the previous documentation time for the comparison of the physical glass slides and respective tissue blocks took around minutes or even hours for the pathologists and technicians. The introduction of BlocDoc allowed a significant reduction of the time required for this task thanks to the possibility of visualizing the tissue block scans directly from the pathologists’ workstations rather than having to manually search and retrieve the tissue blocks themselves from the archive. The average turnaround time is significantly shortened by the digital transition. While apparently more time is required for each case (e.g., scanning the slides before delivering them generates a small delay compared with direct delivery to the pathologist), this is more than balanced by the savings in hands-on time (that is freed up for other tasks) and by the reduction of mistakes, variability and uncertainty in the tissue processing steps.

The completely agnostic approach to the digital pathology workflow is another important point to underline. We believe this is a strong point of our approach to implement the digital workflow. Interoperability is of paramount importance when implementing a digital workflow with the possibility to use WSI for primary histological diagnosis. Thanks to the use of a standard communication approach (i.e., HL7 communication standard) it will be possible not only to interface different machines (different printers for slides, blocks, labels, stainers, immunostainers, etc.) to the LIS, but also to integrate different scanners from different vendors.

The interoperability, together with the lean approach, gives to the path lab the possibility to implement the digital workflow in a very smooth way, without being tied to a single vendor. This is also in line with the possibility of a dynamic implementation of the automation or other things.

The dramatic changes of the Anatomic Pathology in Caltagirone significantly impacted even on the structural disposition of the instruments and offices. This eventually allowed us to re-allocate some spaces to new applications (e.g., obtaining the molecular pathology section previously absent in the lab), as well as significantly reducing the transfer of material and personnel around the laboratory, resulting in time- and cost-effectiveness.

This is further stressed by the relatively low impact of the different novelties introduced (e.g., laboratory reorganization, LIS, slide scanners, dedicated computers/screens, software, storage system. trained personnel) on the overall costs of the department. In Italy, it is customary to rent rather than buy instruments, so for example an expensive slide scanner impacts on the lab balance for only approximately 5000 €/month. The costs of storage have been discussed earlier, and regarding computers, we show that mid-level computers with ordinary monitors (500–600€ total) are adequate for WSI viewing and LIS operations.

Skepticism of technicians towards DP is often cited as a problem to overcome. We found that some features of the new workload are actually preferred by the technicians, if compared to the old workflow. Examples include having 2D barcodes printed directly onto blocks and slides with no need of handwriting, digitizing glass slides by simply loading a scanner with no need to assemble and deliver them, and archiving blocks and glass slides by using computers instead of wasting time to put everything in numerical order. This also demonstrates that the digital workflow corresponds to a decrease in workload for the technicians which is in contrast with the idea of additional workload.

Finally, the adoption of computer-aided diagnostic and artificial-intelligence tools is allowing the construction of a digital hub based in Caltagirone (“House of the Science”) that will coordinate the widest renal pathology network in Italy, collecting cases from an already established nephropathology service in the North of the country that migrated all the routine renal biopsy diagnoses to WSI in 2014 [32]. This will further guarantee an equal access to the best diagnostic renal pathology services without the need to move patients, glass slides or paraffin blocks around Italy, additionally constructing a repository of non-neoplastic renal diseases that can serve as an educational atlas as well as a research database.

## 5. Conclusions

Based on the previous “Catania experience”, the implementation of a fully digital workflow in the Gravina Hospital of Caltagirone was possible and easy to achieve in about 4 months. Following the step-by-step instructions, the implementation of a paperless routine with more standardized and safe processes, the possibility to manage the priority of the cases and to implement artificial intelligence (AI)-tools are no more an utopia for every “analogic” pathology department. Digitization of the slides is only the last step of the “digital workflow” that aims to achieve safety and efficiency for pathologists and patients. Our hope and vision is that ALL labs will switch to this digital workflow believing that this will become the standard of care in pathology, for a matter of ethics more than economics.

## Figures and Tables

**Table 1:** Automation introduced at every step of the workflow.

Phase	Automation Introduced
Accessioning	Adoption of order entry
	A4 flat scanner to digitize all the paper documents associated with the cases (i.e., endoscopic exams, clinical annotations, etc.)
Grossing	Introduction of a laser block printer at grossing
	Introduction of a camera device to take pictures at the grossing bench Possibility to capture the material in the block
Processing/ embedding	Possibility of matching blocks produced at grossing with those sent to processing by using a real-time multi-barcode scanner
Sectioning	Possibility to capture the cut surface of the block for review purposes
	Automated printing of barcodes directly on glass slides rather than on labels
Staining	Automation of requests of histochemical and immunohistochemical stains, which are delivered directly to the stainer
Archiving	Improvement of the archiving of slides and blocks, whose position in the storage trays is random and tracked automatically by barcode scanning

**Table 2:** The different computer devices employed in the Caltagirone digital pathology lab for the pathologists’ workstations. CPU, central processing unit; RAM, random-access memory; OS, operating system; W10, Windows 10 (Microsoft, Redmond, WA, USA).

CPU	Clock Speed	RAM	OS	Dedicated Video
AMD Ryzen 5Pro 2400 G	3.60 GHz	8 GB	W10 64 bit	none (CPU-integrated)
Intel Core i3-9100	3.60 GHz	8 GB	W10 64 bit	none (CPU-integrated)
Intel Core i7-8700	3.20 GHz	8 GB	W10 64 bit	none (CPU-integrated)
Intel Core i5-4590	3.30 GHz	8 GB	W10 64 bit	none (CPU-integrated)
Intel Core i3-2120	3.30 GHz	4 GB	W10 64 bit	none (CPU-integrated)
Intel Celeron 3865U	1.80 GHz	8 GB	W10 64 bit	none (CPU-integrated)

**Table 3:** Specifications of monitors used for WSI visualization.

Manufacturer	Size	Resolution	Type	Refresh Rate (Hz)
Hannstar	23.6 inch	1920 × 1080 pixels	LCD	60
Philips	27 inch	1920 × 1080 pixels	LED	75
Fujitsu	27 inch	2560 × 1440 pixels	LCD	60

**Table 4:** Different steps of specimen processing as they are performed before and after the implementation of digital pathology in the laboratory. DP, digital pathology; LIS, laboratory information system; WSI, whole slide image.

	Before DP Implementation	After DP Implementation
Accessioning checkpoint	Paper request with handwritten patient and specimen data	Order entry system, barcode identifying patient, case, and specimen container, information imported from the integrated hospital LIS with digital request (no more transcription errors)
	Manual check for correspondence between request paper and label on the specimen	Progressive number linked to the barcode generated and used for all sorts of assets generated for that case (tracking of the sample through its journey in the laboratory)
	Manual insertion of the case in the AP-LIS or (worse) new internal “working paper” generated to accompany the specimen in the different subsequent phases of the process (lab sheet)	The administrative will take a picture of the container and of the specimen and those photos will be attached to the case file (medico-legal registry)
	-	Documents attached to the specimen are scanned and attached to the case file (relevant information handy)
Grossing checkpoint	The grossing operator (e.g., pathologist) has the working paper (lab sheet) as the only reference to the case	Automatic access to the case by scanning the identification barcode on the sample container
	No pictures of the sample as it is when it arrives at the grossing room are taken.	Photographic documentation of different grossing steps (specimen in the container, during grossing and within the cassettes) guarantees the preservation of the case features and identification
	Manual transcription of macroscopic description of the sample by the pathologist or the assistant technician (dictation/transcription errors)	Direct dictation of the macroscopic description of the sample converted to text through voice recognition functions of the LIS
	Cassettes are labeled manually by the pathologist/technician	Cassettes are printed with the identification code of the sample to be tracked in further workstations
Sectioning checkpoint	The number transcribed by the grossing operator on the block is copied on the slide, possible source of errors	The code printed on the paraffin block may be scanned to open the case file through the integrated LIS preventing transcription errors
	The needed stain for each case is reported on the working paper (lab sheet) or indicated by the color of the cassette	The technician can check how many and which kind of slides are needed for each block directly on the LIS
	The generated slides are manually transcribed by the sectioning technician, no barcode is printed on the slide	For each paraffin block, one or more printed glass slides are generated through a dedicated printer, including the identification code
	After the sectioning phase, the block is archived and no pictures of the cut surface of the block are taken	After sectioning, each paraffin block may be photographed to assess whether all the material emerged on the glass slide/WSI
	The sectioning phase lack strict quality criteria, the presence of artifacts, folding, inappropriate coverslipping does not significantly impair the physical microscope visualization	Sectioning phase should follow high operative standards, reducing the risk of artifacts that can impair the scanning phase

## Data Availability

All data generated or analyzed during this study are included in the article.
